# A simple and nondestructive approach for the analysis of soluble solid content in citrus by using portable visible to near‐infrared spectroscopy

**DOI:** 10.1002/fsn3.1550

**Published:** 2020-04-14

**Authors:** Pao Li, Shangke Li, Guorong Du, Liwen Jiang, Xia Liu, Shenghua Ding, Yang Shan

**Affiliations:** ^1^ College of Food Science and Technology Hunan Provincial Key Laboratory of Food Science and Biotechnology Hunan Agricultural University Changsha China; ^2^ Hunan Agricultural Product Processing Institute Hunan Academy of Agricultural Sciences Changsha China; ^3^ Beijing Work Station Technology Center Shanghai Tobacco Group Co. Ltd Beijing China

**Keywords:** chemometric method, citrus soluble solid content, nondestructive, portable visible to near‐infrared spectroscopy, variable selection

## Abstract

A simple and nondestructive method for the analysis of soluble solid content in citrus was established using portable visible to near‐infrared spectroscopy (Vis/NIRS) in reflectance mode in combination with appropriate chemometric methods. The spectra were obtained directly by the portable Vis/NIRS without destroying samples. Outlier detection was performed by using leave‐one‐out cross‐validation (LOOCV) with the 3σ criterion, and the calibration models were established by partial least squares (PLS) algorithm. Besides, different data pretreatment methods were used to eliminate noise and background interference before calibration, to determine the one that will lead to better model accuracy. However, the correlation coefficients are all <0.62 and the results of all pretreatments are still unsatisfactory. Variable selection methods were discussed for improving the accuracy, and variable adaptive boosting partial least squares (VABPLS) method was used to get higher robustness models. The results show that standard normal variate (SNV) transformation is the best pretreatment method, while VABPLS can significantly simplify the calculation and improve the result even without pretreatment. The correlation coefficient of the best prediction models is 0.82, while the value is 0.48 for the raw data. The high performance shows the feasibility of portable Vis/NIRS technology combination with appropriate chemometric methods for the determination of citrus soluble solid content.

## Graphical abstract

A simple and nondestructive method for the analysis of citrus sugar content was established using portable visible to near‐infrared spectroscopy in reflectance mode in combination with appropriate chemometric methods. SNV transformation is the best pretreatment method. VABPLS method can significantly simplify the calculation and improve the results even without pretreatment.

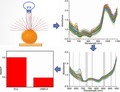

## 1 INTRODUCTION

Citrus industry is the largest fruit industry in the world, and China is a big country of citrus production. There are significant differences in citrus qualities among different varieties and geographical origins. However, the quality analysis technology of citrus mainly focuses on external quality detection, such as weight and color, which is susceptible to subjective factors. Sugar content of citrus is the main characteristic index to evaluate the internal quality of citrus fruits. Chemical titration method (Marrubini, Papetti, Genorini, & Ulrici, 2016), high‐performance liquid chromatography (HPLC) using electrochemical detectors (Švecová, Bordovská, Kalvachová, & Hájek, 2015), UV‐Vis detector (Aires et al., 2017), fluorescence detector after precolumn derivatization (Masuda, Kaneko, & Yamashita, 2010), and tandem mass spectrometry (Shindo et al., 2013) have been reported for analyzing sugar content and composition in fruits. However, chemical titration methods demand time‐consuming operations for sample preparation, while chromatographic methods generally demand expensive equipment and solvent elution. Sugar content represented by soluble solid content is the most important quality index for citrus industry to determine marketing standards (Jin Lee et al., 2004; Marrubini et al., 2016; Švecová et al., 2015). A soluble solid content analyzer, named saccharimeter, was developed by measuring the refractive index or polarization rotation angle of optically active sugars, which is widely used in fruit and wine processing industries (Jin Lee et al., 2004). The method, however, still requires sample destruction and is time‐consuming. Low cost, nondestructive, and accurate analysis of soluble solid content in citrus has become a new trend in citrus production area.

Visible to near‐infrared spectroscopy (Vis/NIRS) is a simple, fast, and nondestructive analytical technique, which is widely used in the analysis of complex samples in food (Towett et al., 2013; Zhu, Chen, Wu, Xing, & Yuan, 2018), agriculture (Tardaguila, Fernández‐Novales, Gutiérrez, & Paz Diago, 2017), and medicine industries (Li, Du, Cai, & Shao, 2012). In recent years, the development trend of Vis/NIRS instruments is miniaturization and low manufacturing cost. Various portable Vis/NIRS instruments have been developed (Cirilli et al., 2016). However, due to the miniaturization of instruments, there are many deficiencies in spectral resolution, scanning range, sensitivity, long‐term stability, reliability, accuracy, and instrument standardization of portable Vis/NIRS instruments. Besides, due to the low sensitivity and complexity of the samples, the useful information is usually carried by a broad spectral peak. In order to solve the problems, a large number of chemometric methods have been developed. Partial least squares (PLS) regression and related robust techniques are the most commonly used methods for establishing quantitative models (De Luca et al., 2019; Li, Shao, & Cai, 2007; Sampaio et al., 2018). Furthermore, a large number of spectral pretreatment methods for baseline correction and background removal were developed, while each possesses advantage and drawbacks (Bian, Li, Shao, & Liu, 2016; Han, Huang, et al., 2017; Shao, Bian, & Cai, 2010). It is very important to choose the proper pretreatment method, which can improve the accuracy of quantitative analysis model to a certain extent. Besides, poor models may be obtained when the spectra contain nonmodeled information. To solve this problem, variable selection methods such as Monte Carlo uninformative variable elimination (MC‐UVE) (Cai, Li, & Shao, 2008), randomization test (RT) (Xu, Liu, Cai, & Shao, 2009), competitive adaptive reweighted sampling (CARS) (Li, Liang, Xu, & Cao, 2009), and related techniques (Han, Tan, et al., 2017) were proposed for building robust and accurate models. In our previous work, variable adaptive boosting partial least squares (VABPLS) (Li, Du, Ma, Zhou, & Jiang, 2018) was proposed to obtain robustness models and improve the prediction ability by simultaneous weighting samples and variables in the boosting step.

Vis/NIRS has attracted more and more attention due to its fast and nondestructive characteristics in the analysis of soluble solid content in citrus (Cavaco et al., 2018; Cayuela & Weiland, 2010; Cen, He, & Huang, 2007). The prediction of soluble solid content in citrus by Vis/NIRS and sensory test was investigated and the result shows that the nondestructive method can meet the sensory requirements of consumers (Yuan et al., 2013). The sample temperature affects the spectrum in a nonlinear way. To solve the problem, global temperature calibration model of Fourier transform near‐infrared reflectance (FT‐NIR) spectroscopy was developed and has been used successfully to measure soluble solid content in citrus (Lu et al., 2006). However, the peel of citrus has great interference to the spectra. In addition, most portable Vis/NIRS was grating scanning one, which is different from FT‐NIR spectroscopy with good detection results. Noise, background, and nonmodeled information interference are unavoidable in portable Vis/NIRS signals. At present, there is little research on the application of portable Vis/NIRS in citrus soluble solid content analysis.

The aim of this study is to establish appropriate chemometric methods for portable Vis/NIRS instruments to obtain reliable and accurate results of citrus soluble solid content determination. Different pretreatment methods were analyzed, while variable selection methods and VABPLS method were investigated to get higher robustness models. Correlation coefficient of cross‐validation (RCV) and root mean square error of cross‐validation (RMSECV) were applied to evaluate the performances of the final models, while correlation coefficient (R) and root mean square error of prediction (RMSEP) were used to evaluate the methods. Furthermore, the selected characteristic wavelengths were also discussed in detail. Based on portable Vis/NIRS and chemometric methods, the technology can be regarded as a simple, low cost, nondestructive, and accurate way for the analysis of citrus soluble solid content, which can be applied in future fruit production.

## 2 MATERIALS AND METHODS

### 2.1 Citrus sample

In this study, 105 *Citrus sinensis* (L.) Osbeck samples of uniform color (orange), shape, and size (~60 mm diameter) were randomly purchased from local supermarkets between November and December. To reduce the effect of sample temperature on the prediction accuracy, the samples were placed at room temperature for 24 hr for equilibration. Then, the samples were cleaned and numbered before measurement.

### 2.2 Citrus soluble solid content determination

Soluble solid contents were measured from squeezed‐out juices of the samples by digital refractometer saccharimeter (model PR‐101, Atago Co. Ltd.) and were provided by Beijing Weichuangyingtu Technology Co., Ltd. (Jin Lee et al., 2004). Each content was averaged from three parallel measurements.

### 2.3 Instrumentation and measurements

Vis/NIRS spectra were obtained by a NIRMagic 1,100 spectrometer (Beijing Weichuangyingtu Technology Co., Ltd) combining a standard multichannel grating detector in the diffuse reflectance mode with integrating sphere diffuse reflection accessory. The power of light source was 12 V/20 W, while the integration time and average time were 40 ms and 2s. The white reference and dark reference were collected for each collection. The citrus was placed directly in the center of the spot. The spectra were collected at the equator location, and the average of four equator locations was used. Each spectrum is composed of 501 data points recorded from 600 to 1,100 nm and is averaged from three parallel measurements.

### 2.4 Data analysis

Outlier detection was performed by using leave‐one‐out cross‐validation (LOOCV) with the 3σ criterion. Kennard–Stone (KS) method was used for the partition of the calibration and test set, and the calibration models were established by PLS algorithm. The performances of the developed models were evaluated in terms of RCV and RMSECV, while the prediction performances were evaluated by R and RMSEP. To some extent, the robustness of the model can be proved with the method. Monte Carlo cross‐validation (MCCV) with adjusted Wold's R criterion was used for determination of latent variable (LV) number. Besides, to eliminate noise and background interference, the spectra were treated by different pretreatment methods, such as bias correction, detrend, standard normal variate (SNV) transformation, maximum and minimum normalization, multiplicative scatter correction (MSC), first‐order derivative (1st) and second‐order derivative (2nd), continuous wavelet transform (CWT), and their combinations, to obtain reliable quantitative calibration models.

Generally, several hundreds or even thousands of variables (wavelength) can be obtained in a spectrum. Some of the variables may contribute more collinearity and noise than relevant information to models (Li et al., 2009; Xu et al., 2009). Poor models may be obtained when the spectra contain nonmodeled information. Therefore, variable selection methods such as MC‐UVE and CARS were used for building parsimonious and accurate models. The former method builds a large number of models with randomly selected calibration samples, and then, the wave numbers are evaluated with a parameter of stability. The larger the stability is, the more significant the wave number will be (Cai et al., 2008). The latter method mimics the “survival of the fittest” principle which is the basis of Darwin's Evolution Theory and has been successfully adopted to select the key wavelengths (Li et al., 2009). Besides, VABPLS was applied to get higher robustness models and enhance the prediction ability by simultaneous weighting of samples and variables in the boosting step (Li et al., 2018). Furthermore, consensus partial least squares regression (cPLS) and boosting PLS with the same training set and prediction set were used as comparison.

The programs were performed using Matlab 8.3 (The Mathworks) and run on a personal computer.

## 3 RESULTS AND DISCUSSION

### 3.1 Correlation between spectra and citrus soluble solid contents

**Figure 1:**
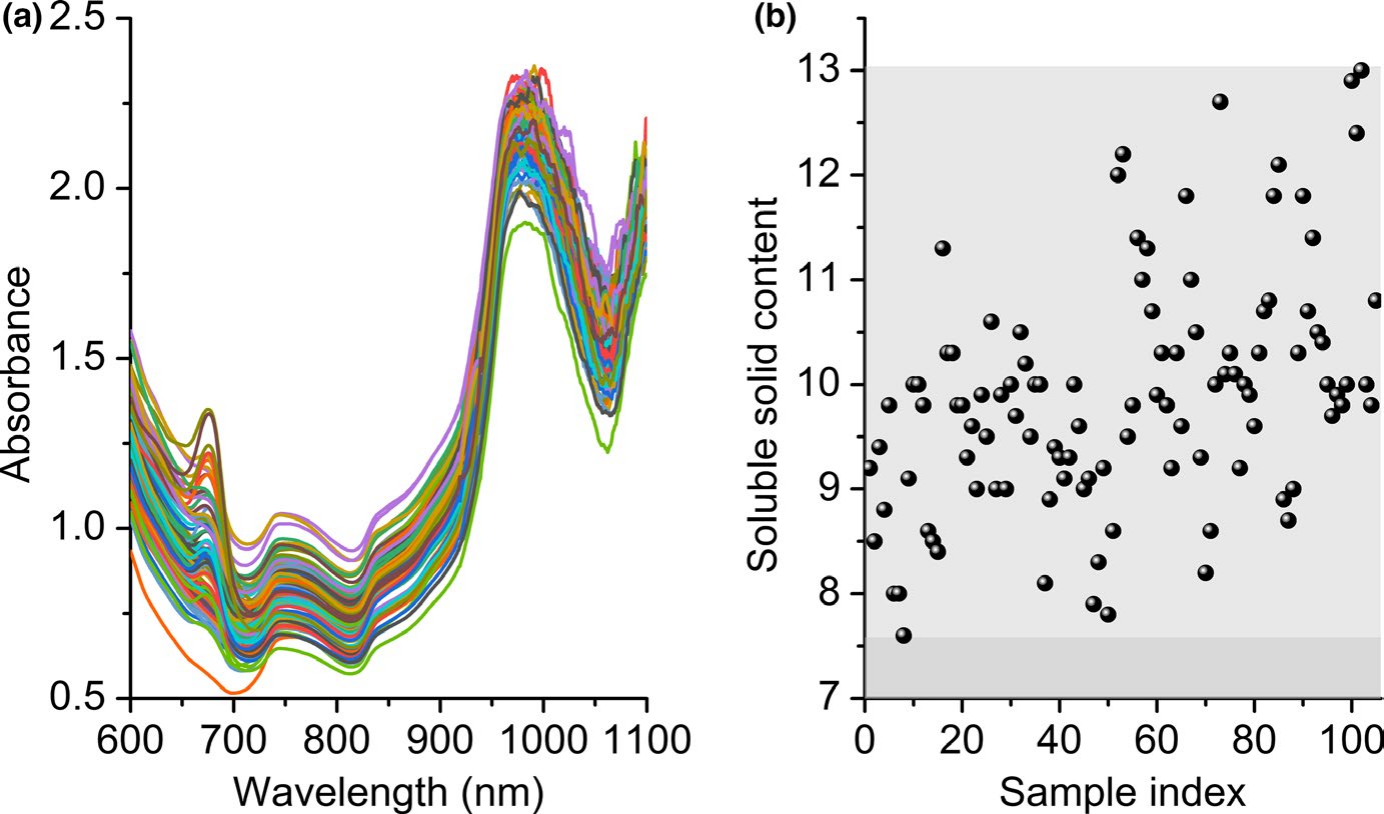
Original spectra for the citrus dataset (a) and distribution of soluble solid contents (b)

**Figure 2:**
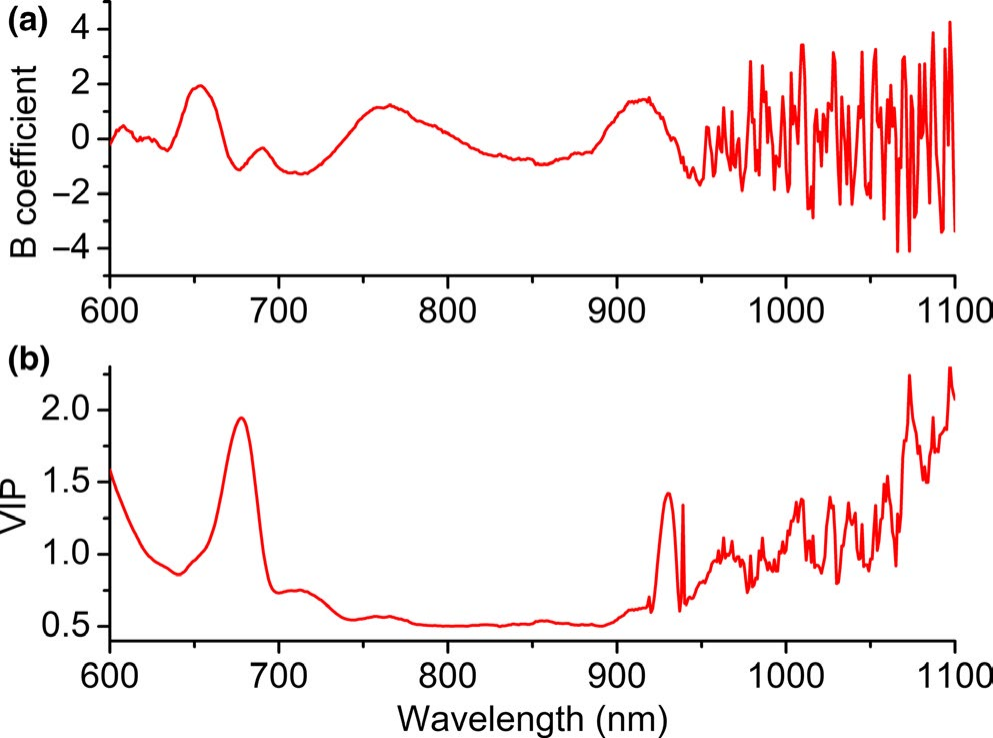
B coefficients (a) and variable importance in the projection (VIP) values (b)

Figure 1 shows the original spectra for the citrus dataset and distribution of soluble solid contents. It can be seen that obvious peaks around 680 and 750 nm in the spectra. Obvious noise interference exists at the range above 950 nm. Therefore, the spectra in the range of 600 to 950 nm were selected for the further calculation, which is consistent with previous report (Jin Lee et al., 2004). Besides, it can be seen that there is interference of baseline drift in the original spectra. It is not feasible to use the original spectra directly for the analysis of soluble solid content. Information about orange color is found at 650–700 nm range of the visible spectrum. A continuous increase in absorbance was observed from 710 to 990 nm. The peaks around 760 and 970 nm were normally attributed to water or OH groups. Furthermore, from Figure 1b, the soluble solid content of all samples ranged from 7.5 to 13 °Brix. Figure 2 shows B coefficients and variable importance in the projection (VIP) values. It can be seen that there is also obvious noise interference above 950 nm, and thus, the range of 600 to 950 nm was selected for the further calculation. A high regression coefficient can be found in the wavelengths of 640–700 and 890–940 nm, indicating the significance of these wavelengths.

### 3.2 Outlier detection

**Figure 3:**
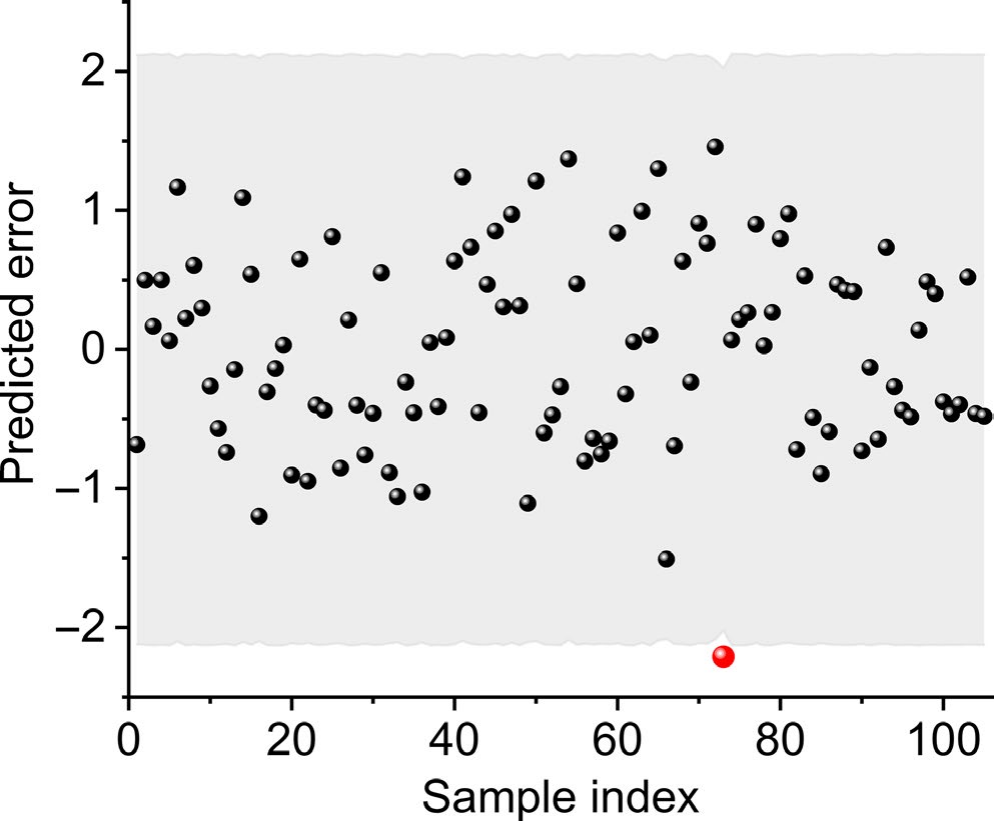
Distribution of the prediction errors and 3σ criterion

Outliers may be caused by instability of instruments and operational errors, which may reduce the quality of the model. In this paper, the outlier detection was performed by using LOOCV with the 3σ criterion. Figure 3 shows a plot of the prediction errors and the 3σ criterion. It is clear that the value of sample no. 73 was out of the threshold, which was considered to be an outlier.

### 3.3 Effect of pretreatment

A total of 104 citrus samples were divided into a calibration dataset with 69 samples and a test dataset with 35 samples by KS method. In addition, 100 random grouping results with the same sizes of calibration and test dataset were used as comparison. The calibration model was established by PLS algorithm, and MCCV with adjusted Wold's R criterion was used for determination of LV number. In order to build an optimal model, in this paper, the spectra were treated by different pretreatment techniques, such as bias correction, detrend, SNV transformation, maximum and minimum normalization, MSC, 1st, 2nd, CWT, and their combinations to obtain reliable quantitative calibration models. Table 1 shows a comparison of the LV, RMSECV, RCV, RMSEP, and R with the thirteen pretreatment methods. Compared with the results of the raw spectra in the range of 600–1100 nm, the effect of pretreatment methods has not been significantly improved. The optimal LV number is more than 10 with 1st‐DT method. The R values are all less than 0.62, and the results of all pretreatments are still unsatisfactory.

**Table 1:** Comparison of the LV, RMSECV, RCV, RMSEP, and R by different pretreatment methods with the full spectra and spectra in the range of 600 to 950 nm

	Method name	LV	RMSECV	RCV	RMSEP	R
Full spectra	Raw spectra	5	0.854	0.691	0.803	0.487
	De Bias	4	0.794	0.735	0.728	0.595
	Detrend	3	0.828	0.706	0.756	0.552
	SNV	3	0.814	0.719	0.760	0.546
	Min Max	3	0.808	0.723	0.732	0.561
	MSC	3	0.812	0.720	0.759	0.547
	1st	6	0.955	0.619	0.940	0.372
	2nd	2	1.206	0.200	1.295	−0.164
	1st‐DT	13	1.118	0.524	0.922	0.371
	1st‐SNV	6	0.977	0.603	0.982	0.331
	1st‐MSC	6	0.964	0.611	0.981	0.323
	CWT	6	0.970	0.608	0.921	0.397
	CWT‐MSC	6	0.961	0.615	0.960	0.353
	CWT‐SNV	6	0.972	0.607	0.961	0.360
Spectra in the range of 600 to 950 nm	Raw spectra	9	0.683	0.800	0.662	0.778
	De Bias	10	0.663	0.813	0.599	0.814
	Detrend	7	0.637	0.825	0.617	0.803
	SNV	9	0.673	0.805	0.595	0.814
	Min Max	9	0.673	0.809	0.600	0.810
	MSC	7	0.665	0.809	0.653	0.777
	1st	7	0.681	0.798	0.664	0.770
	2nd	8	0.868	0.664	0.839	0.661
	1st‐DT	7	0.707	0.779	0.739	0.727
	1st‐SNV	7	0.680	0.800	0.648	0.785
	1st‐MSC	6	0.670	0.804	0.666	0.772
	CWT	7	0.679	0.800	0.660	0.772
	CWT‐MSC	6	0.672	0.803	0.655	0.777
	CWT‐SNV	8	0.677	0.803	0.654	0.786

Acceptable results cannot be obtained by the models directly built with the full spectra. This poor result may be caused by many reasons. One crucial reason of them is that the noise interference exists in the range above 950 nm. The comparison of the LV, RMSECV, RCV, RMSEP, and R with the spectra in the range of 600 to 950 nm was also shown in Table 1. It is clear that the optimal LV numbers are all less than 10, which are more reliable than the results with full spectra. The results of pretreatment methods are slightly better than those of the raw spectra except the 2nd method, and the combinations of pretreatment methods cannot further improve the RMSECV values. SNV is the best pretreatment method, and R value is as high as 0.814. Due to the irregular surface, the spectrum of citrus sample is easily affected by light scattering. The interferences of solid particle size, surface scattering, and the change of optical path of diffuse reflection spectra can be eliminated by SNV method. The results are consistent with the related reference (Cavaco et al., 2018).

### 3.4 Variable selection

Variable selection can be used to further optimize the model of Vis/NIR quantitative analysis. Variable selection methods, such as MC‐UVE and CARS, were used for improving the accuracy in this study. Besides, VABPLS method was used to get higher robustness models and enhance the prediction ability. Furthermore, the results of cPLS and boosting PLS with the same training set and prediction set were also obtained as comparison. A total of 100 independent runs were performed, and the means of numbers of variables, RMSEPs, and Rs are obtained. The performance of the final models was evaluated according to the RMSEP and R with the test set.

**Figure 4:**
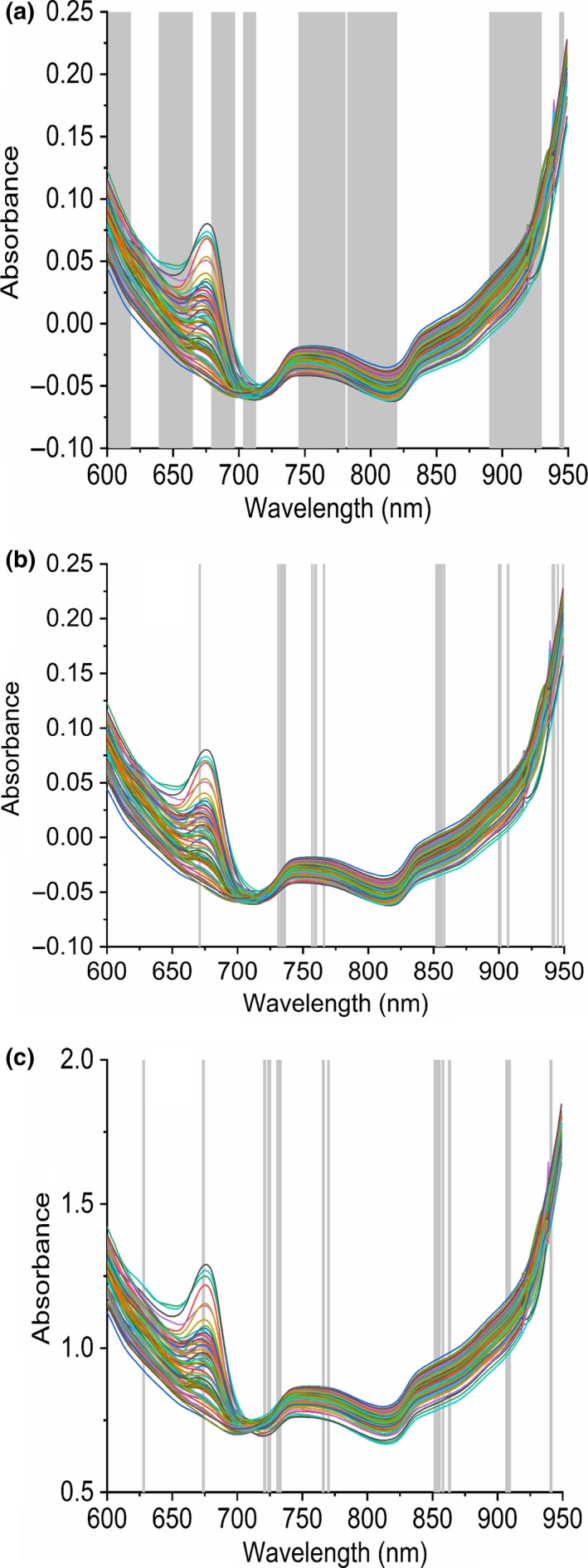
Variable distribution of MC‐UVE and the SNV methods (a), variable distribution of CARS and SNV methods (b), and variable distribution of VABPLS and SNV methods (c)

The PLS models developed with MC‐UVE are shown in Table 2. Compared with the raw spectra‐PLS model, the numbers of variables decreased from 350 to 91 with the CWT‐SNV method. SNV is the best pretreatment method, and R value is as high as 0.80. Figure 4a shows the variable distribution with MC‐UVE and the SNV methods. Variable selection can not only simplify the model, but also extract the wavelengths related to the components. Therefore, the wavelengths which are less interfered by orange peel can be obtained. As a consequence, mainly eight wavelength bands were retained. They were 600–617 nm, 639–665 nm, 678–695 nm, 703–713 nm, 745–780 nm, 782–819 nm, 890–930, and 943–947 nm, which belong to red‐orange absorption band, OH third and second overtone bands, and CH third overtone band. The bands are consistent with the result for the analysis of soluble solid content in pear (Xu, Qi, Sun, Fu, & Ying, 2012). However, the numbers of variables for the raw spectra or the spectra using De Bias, Min Max, 1st, 2nd, and CWT methods are as high as 340, and the variable selection was rather unsatisfactory. As a result, the RMSEPs and Rs of the raw spectra by MC‐UVE method are nearly the same as the results without variable selection.

**Table 2:** Results with the spectra in the range of 600 to 950 nm by different pretreatment methods and variable selection, compared with cPLS and boosting PLS methods

Variable selection	Method name	LV	Variables	RMSEP^a^	σ(RMSEP)^b^	R^a^	σ(R)^b^
MC‐UVE	Raw spectra	9	340	0.661	0.000	0.779	0.000
	De Bias	10	340	0.607	0.000	0.809	0.000
	Detrend	7	140	0.639	0.002	0.794	0.001
	SNV	9	180	0.611	0.003	0.802	0.002
	Min Max	9	340	0.607	0.001	0.806	0.001
	MSC	7	100	0.681	0.022	0.756	0.016
	1st	7	340	0.664	0.001	0.771	0.000
	2nd	8	340	0.855	0.001	0.648	0.000
	1st‐DT	7	119	0.730	0.022	0.734	0.014
	1st‐SNV	7	145	0.646	0.013	0.789	0.007
	1st‐MSC	6	179	0.640	0.009	0.788	0.005
	CWT	7	340	0.662	0.000	0.770	0.000
	CWT‐MSC	6	102	0.714	0.014	0.735	0.012
	CWT‐SNV	8	91	0.743	0.005	0.731	0.003
CARS	Raw spectra	9	81	0.634	0.022	0.799	0.014
	De Bias	8	40	0.611	0.025	0.800	0.016
	Detrend	5	28	0.607	0.019	0.819	0.010
	SNV	8	39	0.592	0.026	0.821	0.015
	Min Max	5	40	0.635	0.032	0.773	0.018
	MSC	6	24	0.603	0.028	0.797	0.019
	1st	6	34	0.643	0.032	0.798	0.020
	2nd	6	43	0.778	0.050	0.740	0.048
	1st‐DT	5	34	0.710	0.024	0.743	0.013
	1st‐SNV	1	17	0.674	0.049	0.759	0.027
	1st‐MSC	6	29	0.651	0.045	0.732	0.026
	CWT	5	34	0.659	0.027	0.770	0.020
	CWT‐MSC	1	12	0.650	0.018	0.804	0.010
	CWT‐SNV	5	9	0.691	0.048	0.773	0.027
VABPLS	Raw spectra	9	67	0.596	0.025	0.820	0.016
	De Bias	10	33	0.600	0.025	0.814	0.015
	Detrend	7	74	0.571	0.010	0.814	0.006
	SNV	9	50	0.579	0.033	0.824	0.019
	Min Max	9	39	0.602	0.026	0.799	0.017
	MSC	7	45	0.566	0.021	0.814	0.011
	1st	7	33	0.643	0.013	0.787	0.008
	2nd	8	26	0.862	0.072	0.700	0.063
	1st‐DT	7	42	0.691	0.021	0.756	0.012
	1st‐SNV	7	63	0.619	0.020	0.770	0.011
	1st‐MSC	6	63	0.613	0.012	0.781	0.008
	CWT	7	28	0.654	0.013	0.778	0.008
	CWT‐MSC	6	49	0.615	0.015	0.787	0.009
	CWT‐SNV	8	69	0.616	0.013	0.804	0.008
cPLS	Raw spectra	9	350	0.643	0.006	0.791	0.030
	De Bias	10	350	0.599	0.006	0.815	0.032
	Detrend	7	350	0.627	0.004	0.798	0.020
	SNV	9	350	0.592	0.006	0.817	0.032
	Min Max	9	350	0.612	0.007	0.803	0.031
	MSC	7	350	0.636	0.006	0.787	0.025
	1st	7	350	0.665	0.005	0.767	0.018
	2nd	8	350	0.758	0.011	0.672	0.022
	1st‐DT	7	350	0.739	0.004	0.721	0.015
	1st‐SNV	7	350	0.637	0.005	0.786	0.020
	1st‐MSC	6	350	0.632	0.005	0.787	0.018
	CWT	7	350	0.661	0.004	0.769	0.017
	CWT‐MSC	6	350	0.631	0.004	0.788	0.018
	CWT‐SNV	8	350	0.630	0.005	0.794	0.023
Boosting PLS	Raw spectra	9	350	0.633	0.006	0.798	0.026
	De Bias	10	350	0.612	0.008	0.807	0.032
	Detrend	7	350	0.605	0.003	0.813	0.018
	SNV	9	350	0.593	0.007	0.817	0.036
	Min Max	9	350	0.632	0.007	0.793	0.031
	MSC	7	350	0.621	0.003	0.798	0.014
	1st	7	350	0.651	0.004	0.778	0.013
	2nd	8	350	0.781	0.018	0.653	0.034
	1st‐DT	7	350	0.708	0.004	0.745	0.015
	1st‐SNV	7	501	0.613	0.004	0.801	0.016
	1st‐MSC	6	501	0.619	0.003	0.799	0.012
	CWT	7	501	0.650	0.004	0.780	0.015
	CWT‐MSC	6	501	0.613	0.002	0.802	0.010
	CWT‐SNV	8	501	0.595	0.007	0.813	0.030

^a^ RMSEP and R are the average value obtained by 100 runs, respectively.

^b^ σ(RMSEP) and σ(R) are the standard deviation of RMSEP and R obtained by 100 runs, respectively.

The PLS models developed with CARS are shown in Table 2. Compared with the raw spectra‐PLS model, the numbers of variables with CARS method decreased from 350 to 81. The results of the raw spectra by CARS methods are better than those without variable selection. The results of most pretreatment methods are better than those of the raw spectra, and the combinations of pretreatment methods can also improve the RMSECV values. SNV is still the best pretreatment method, and R value is as high as 0.821. Figure 4b shows the variable distribution with CARS and SNV methods. As a consequence, mainly eight wavelength bands were retained. They were 627–629 nm, 673–675 nm, 720–726 nm, 730–734 nm, 765–771 nm, 851–864 nm, 906–910, and 940–942 nm, which belong to red‐orange absorption band, OH third and second overtone bands, and CH third overtone band. However, the number of variables screened by CARS is much smaller than that of MC‐UVE.

VABPLS method was applied to obtain robustness models and improve the prediction ability by simultaneous weighting of samples and variables in the boosting step. The PLS models developed with VABPLS are shown in Table 2. Compared with the raw spectra‐PLS model, the numbers of variables with VABPLS method decreased from 350 to 67. The results show that satisfactory quantitative results can be obtained by VABPLS method even without pretreatment (RMSEP = 0.596, *R* = .820), compared with the results of the raw spectra by CARS methods (RMSEP = 0.634, *R* = .799). Besides, from the tables it can be seen that best model can be obtained by VABPLS method than those of cPLS (RMSEP = 0.592, *R* = .817) and boosting PLS (RMSEP = 0.593, *R* = .817) methods. The result indicates that the variable selection can further improve the model. Simultaneous weighting of sample and variable in the boosting series is more effective than the single weighting sample. Furthermore, SNV is the best pretreatment method (RMSEP = 0.579, *R* = .824), which can correct light scattering properties of the fruit. Satisfactory results can be obtained by most pretreatment methods with VABPLS method.

Figure 4c shows the variable distribution with VABPLS and SNV methods. As a consequence, mainly seven wavelength bands were retained and it was clear that the variable distribution with VABPLS is similar to CARS results. This is because both methods are based on the principle of “survival of the fittest.” The high performance shows the feasibility of portable Vis/NIRS technology combination with appropriate chemometric methods for the determination of citrus soluble solid content.

In order to further verify the accuracy of the developed models, 100 random grouping results with the same sizes of calibration and test dataset of KS method were used as comparison. Table 3 is the results by different modeling methods with the random grouping. Compared with the results with cPLS and boosting PLS methods, the results after variable selection are slightly worse, which might be because the test dataset has values outside the range of calibration dataset. However, among the three variable selection methods, VABPLS method has the best results due to the combination advantages of booting and variable selection.

**Table 3:** Results with the spectra in the range of 600 to 950 nm by different modeling methods with random grouping

Modeling methods	RMSEP^a^	σ(RMSEP)^b^	R^a^	σ(R)^b^
SNV‐PLS	0.7538	0.0921	0.7553	0.0705
SNV‐MC‐UVE‐PLS	0.7759	0.1076	0.7362	0.0872
SNV‐CARS‐PLS	0.8030	0.1219	0.7192	0.1024
SNV‐VABPLS	0.7592	0.1072	0.7524	0.0774
SNV‐cPLS	0.7314	0.0871	0.7653	0.0694
SNV‐Boosting PLS	0.7326	0.0805	0.7668	0.0630

^a^ RMSEP and R are the average value obtained by 100 runs, respectively.

^b^ σ(RMSEP) and σ(R) are the standard deviation of RMSEP and R obtained by 100 runs, respectively.

## 4 CONCLUSION

A simple and nondestructive method for the direct analysis of soluble solid content in citrus was established using portable Vis/NIRS combination with appropriate chemometric methods. Data pretreatment methods can be used to eliminate noise and background interference, while variable selection significantly improves the accuracy. SNV transformation is the best pretreatment method. VABPLS method can significantly simplify the calculation and improve the results. This developed technology based on portable Vis/NIRS and chemometric methods can be regarded as a simple, low cost, nondestructive, and accurate way for the analysis of citrus soluble solid content and can be widely applied in future production. The analysis of different citrus varieties will be considered in the future.

## CONFLICT OF INTEREST

The authors declared that they have no conflicts of interest to this work.

## AUTHOR CONTRIBUTIONS

Pao Li. and Yang Shan contributed to conceptualization; Pao Li. and Yang Shan contributed to methodology; Guorong Du. contributed to software; Pao Li, Shangke Li, and Liwen Jiang contributed to validation; Xia Liu contributed to formal analysis; Pao Li contributed to investigation; Shangke Li contributed to resources; Liwen Jiang contributed to data curation; Pao Li contributed to writing—original draft preparation; Shenghua Ding contributed to writing—review and editing; Shangke Li contributed to visualization; Xia Liu contributed to supervision. All authors have read and agreed to the published version of the manuscript.

## ETHICAL APPROVAL

This study does not involve any human or animal testing.
